# Whole Exome Sequencing of Intermediate-Risk Acute Myeloid Leukemia without Recurrent Genetic Abnormalities Offers Deeper Insights into New Diagnostic Classifications

**DOI:** 10.3390/ijms25168669

**Published:** 2024-08-08

**Authors:** Francesca Guijarro, Sandra Castaño-Díez, Carlos Jiménez-Vicente, Marta Garrote, José Ramón Álamo, Marta Gómez-Hernando, Irene López-Oreja, Jordi Morata, Mònica López-Guerra, Cristina López, Sílvia Beà, Dolors Costa, Dolors Colomer, Marina Díaz-Beyá, Maria Rozman, Jordi Esteve

**Affiliations:** 1 Hematopathology Section, Pathology Department, Hospital Clínic Barcelona, 08036 Barcelona, Spain; scastano@clinic.cat (S.C.-D.); garrote@clinic.cat (M.G.); alamo@clinic.cat (J.R.Á.); magomezh@clinic.cat (M.G.-H.); ilopezor@clinic.cat (I.L.-O.); lopez5@clinic.cat (M.L.-G.); clopez2@clinic.cat (C.L.); sbea@clinic.cat (S.B.); dcosta@clinic.cat (D.C.); dcolomer@clinic.cat (D.C.); mrozman@clinic.cat (M.R.); 2 Institut d’Investigacions Biomèdiques August Pi i Sunyer (IDIBAPS), 08036 Barcelona, Spain; cajimenez@recerca.clinic.cat (C.J.-V.); diazbeya@clinic.cat (M.D.-B.); jesteve@clinic.cat (J.E.); 3 Hematology Department, Hospital Clínic Barcelona, 08036 Barcelona, Spain; 4 Centro Nacional de Análisis Genómico (CNAG), 08028 Barcelona, Spain; jordi.morata@cnag.eu; 5 Biomedical Research Networking Center on Oncology (CIBERONC), 28029 Madrid, Spain; 6 Facultat de Medicina, Universitat de Barcelona, 08007 Barcelona, Spain

**Keywords:** acute myeloid leukemia, intermediate-risk cytogenetics, whole exome sequencing, copy-number analysis, World Health Organization Classification, International Consensus Classification

## Abstract

Two new diagnostic classifications of acute myeloid leukemia (AML) were published in 2022 to update current knowledge on disease biology. In previous 2017-edition categories of AML with myelodysplasia-related changes, AML was not otherwise specified, but AML with mutated *RUNX1* experienced profound changes. We performed whole exome sequencing on a cohort of 69 patients with cytogenetic intermediate-risk AML that belonged to these diagnostic categories to correlate their mutational pattern and copy-number alterations with their new diagnostic distribution. Our results show that 45% of patients changed their diagnostic category, being AML myelodysplasia-related the most enlarged, mainly due to a high frequency of myelodysplasia-related mutations (58% of patients). These showed a good correlation with multilineage dysplasia and/or myelodysplastic syndrome history, but at the same time, 21% of de novo patients without dysplasia also presented them. *RUNX1* was the most frequently mutated gene, with a high co-occurrence rate with other myelodysplasia-related mutations. We found a high prevalence of copy-neutral loss of heterozygosity, frequently inducing a homozygous state in particular mutated genes. Mild differences in current classifications explain the diagnostic disparity in 10% of patients, claiming a forthcoming unified classification.

## 1. Introduction

The World Health Organization (WHO) Classification of Hematolymphoid Tumors allows for a congruent diagnosis across the world since its first edition in 2002. This is a living classification that evolves in accordance with new evidence, increasing our understanding of disease biology. In particular, acute myeloid leukemia (AML) is a highly heterogeneous tumor in which the advent of next-generation sequencing (NGS) has markedly enlarged our knowledge about the genetic lesions involved in its pathogenesis.

In 2022, two parallel classifications appeared: the 5th edition of the WHO Classification (WHO22) [1] and the International Consensus Classification (ICC22) [2]. In both classifications, one of the diagnostic categories that changed the most was AML with myelodysplasia-related changes (AML-MRC), as a consequence of the identification of eight myelodysplasia-related (MR) genes, whose mutations are able to identify secondary AML with high specificity [3]. The revised 4th edition of WHO Classification (WHO17) recognized three possible criteria to diagnose AML-MRC in the absence of recurrent genetic abnormalities and preceding cytotoxic exposure: previous myelodysplastic syndrome (MDS) or myelodysplastic/myeloproliferative neoplasm (MDS/MPN), the presence of any of the defined MDS-related cytogenetic abnormalities, or the presence of multilineage dysplasia (MLD, defined as at least 50% of dysplastic hematopoietic precursors in at least two hematologic lineages). New classifications have eliminated MLD criteria to define AML myelodysplasia-related (AML-MR) but recognize mutations in *ASXL1*, *BCOR*, *EZH2*, *SF3B1*, *SRSF2*, *STAG2*, *U2AF1*, or *ZRSR2* as a novel criteria. Whether this list of genes reflects a true biological subgroup must still be further explored, as other works did not find a strong association between secondary AML and some of them [4] or propose the addition of other genes, such as *RUNX1*, *SETBP1*, and *KMT2A*-PTD [5]. Conflicting results evaluating the prognostic impact of MR mutations in younger patients or de novo AML [5,6,7] also call into question the uniformity of these criteria. Along the same line, the incorporation of this mutational pattern to the adverse-risk group in the last European LeukemiaNet risk classification [8] (ELN) slightly reduced its prognostic accuracy in some works [9,10], indicating they may not constitute a homogeneous subgroup.

In addition, there are some differences between new classifications that deserve clarification. *RUNX1* is a frequently mutated gene that, in the absence of other recurrent genetic alterations or AML-MRC criteria, defined a provisional entity in WHO17 (from now on AML-RUNX1m). This category has been eliminated from new classifications, as this gene was incorporated into the group of MR genes by ICC22, whereas WHO22 does not consider it as such. Furthermore, ontogeny based on clinical criteria still improves prognostication [6], but only WHO22 considers MDS or MDS/MPN history as criteria to diagnose AML-MR.

To address these issues, we have characterized by whole exome sequencing (WES) a cohort of 69 cytogenetic intermediate-risk AML patients without recurrent genetic abnormalities (as defined by WHO17), as they represent a heterogeneous group of patients that have experienced most changes with current classifications. Our aims are to analyze the reallocation of these patients in new 2022 AML entities and to investigate potential clinical and biological heterogeneity through recurrent and less frequent variant assessment and copy-number analysis.

## 2. Results

### 2.1. Patient Characteristics and Their Redistribution in New Diagnostic Categories

Patients were distributed in 27 AML-MRC, 26 AML not otherwise specified (AML-NOS), and 16 AML-RUNX1m, whose clinical characteristics are summarized in Table 1.

No differences regarding sex distribution, age, or white blood cell counts between groups were detected. In contrast, patients diagnosed with AML-RUNX1m showed higher bone marrow (BM) and peripheral blood (PB) blast counts. Cytogenetics results were available for 64 patients. All but one patient with AML-RUNX1m had a normal karyotype (92%), in contrast to 59% of AML-MRC and 62% AML-NOS (*p* = 0.09).

Criteria to define AML-MRC patients were the presence of MLD (*n* = 23), history of MDS (*n* = 11) and/or cytogenetic defining lesions (*n* = 4). In 12 patients, MLD was the single criteria for AML-MRC diagnosis, while all patients with a previous MDS also had MLD. In contrast, all four patients with myelodysplasia-defining cytogenetic lesions did not have any other criteria for AML-MRC diagnosis.

**Figure 1:**
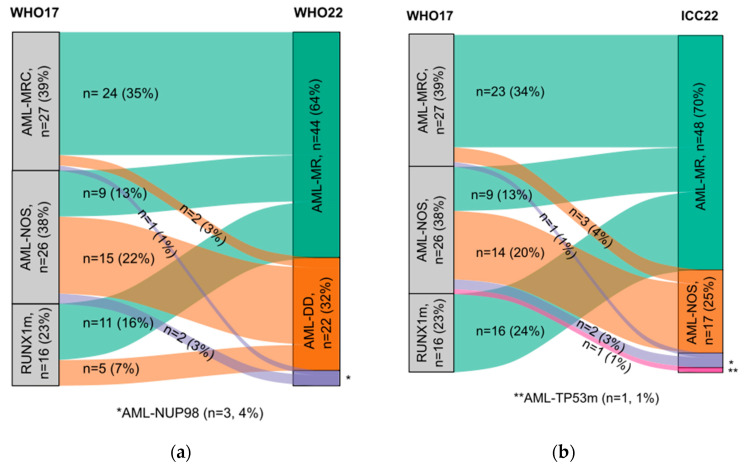
Alluvial plots showing the redistribution of cases according to new classifications. For each diagnostic category (squared boxes), the number of patients following the same classification is indicated in each flow path. (**a**) Reclassification of patients with acute myeloid leukemia (AML) from the World Health Organization Classification of Hematolymphoid Tumors published in 2017 (WHO17) to the version published in 2022 (WHO22); (**b**) reclassification of patients from WHO17 to International Consensus Classification of 2022 (ICC22). Other abbreviations: AML-MRC, AML with myelodysplasia-related changes; AML-NOS, AML not otherwise specified; RUNX1m, AML with *RUNX1* mutation; AML-MR, AML myelodysplasia-related; AML-NUP98, AML with *NUP98* rearrangement; AML-TP53m, AML with *TP53* mutation.

A graphical representation of the redistribution of cases according to new classifications is shown in Figure 1. After applying the WHO22 classification, 56% of patients remained in the same diagnostic category: 15 patients were still diagnosed with AML defined by differentiation (AML-DD, former AML-NOS), and 24 patients corresponded to AML myelodysplasia-related (AML-MR, former AML-MRC). Results were very similar after reclassification of cases according to ICC22, except for two cases: one AML-NOS by WHO17 that was relocated to AML with TP53 mutation (AML-TP53m) and another case with MDS history AML-MR that did not show any MR-genetic alteration (further described below).

With both classifications, two patients with AML-NOS and one patient with AML-MRC defined by MLD would be allocated to the new entity AML with *NUP98* rearrangements (AML-NUP98).

All AML-RUNX1m cases (*n* = 16) were incorporated in AML-MR by ICC22-definition. However, 11 of these cases (69%) also corresponded to AML-MR according to WHO22 because of the co-occurence of other MR-related mutations, being the rest allocated to AML-DD. Moreover, nine AML-NOS cases also reclassified to AML-MR, making AML-MR the most enlarged category due to the high prevalence of MR-mutations.

On the contrary, from 12 patients with MLD as a single criterion for the diagnosis of AML-MRC, only 3 lacked MR mutations (two AML-DD/NOS after reclassification and one AML-NUP98). All three patients presented with megakaryocytic and granulocytic dysplasia; one of them also had erythroid dysplasia. They all had *FLT3*-ITD, either accompanied by *WT1* (*n* = 2) or *KIT* (*n* = 1) mutations.

From 11 patients with history of MDS, two did not have MR-mutations nor myelodysplasia-defining cytogenetic abnormalities. One case was a 41-year-old woman with morphologic dysplasia and long-term cytopenia who had mutations in *FLT3*-ITD, *WT1*, and *RUNX1*. In this case, AML-MR diagnosis did not change because of the history of MDS (WHO22) or because of the presence of a mutation in *RUNX1* (ICC22). The second patient had persistent mild cytopenia and morphologic dysplasia when he was diagnosed with refractory anemia with excess of blasts type 1 (IPSS-risk score of 3.5). Two years later, he developed AML with mutations in *TET2* and *KRAS*. In this case, the different considerations of ontogeny between both classifications allocate him as AML-MR by WHO22, while he would be diagnosed with AML-NOS by ICC22.

### 2.2. Molecular Characterization

#### 2.2.1. Somatic Variants

**Figure 2:**
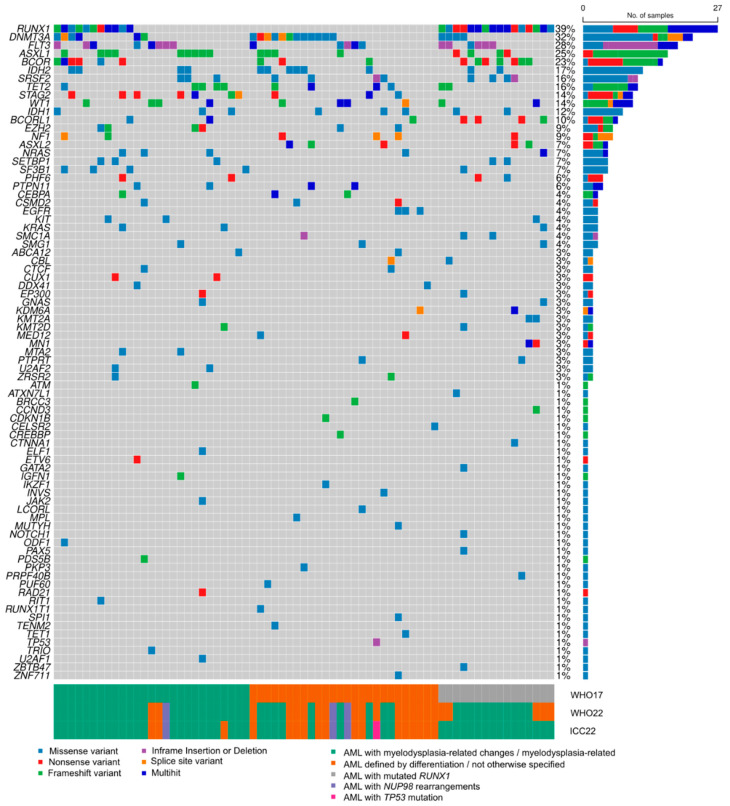
Oncoplot of all variants found in 79 genes in the whole cohort (*n* = 69), including variants of unknown significance, likely oncogenic and oncogenic variants. The panel below shows the correspondence of every case with each diagnostic classification (WHO17, WHO22, and ICC22).

After interrogating 188 genes, we found 339 variants in 79 genes (42%) (Figure 2). These were manually curated, 265 (78%) being classified as oncogenic/likely oncogenic (O/LO) and 74 (22%) as variants of unknown significance (VUS) (Supplementary Table S1). The percentage of VUS variants was higher for low-frequency mutated genes (62% vs. 8%, *p* < 0.001) in comparison to recurrently mutated genes. All patients had at least 1 mutation, with a median number of 5 (range 1–12). The most frequently affected pathways involved activated signaling genes and chromatin modifiers (61% and 59% of samples, respectively), followed by DNA methylation (54%) and myeloid transcription factors (46%). Thirty-five percent of samples had spliceosome mutations, and 22% had mutations in some genes from the cohesin family.

#### 2.2.2. Somatic Oncogenic or Likely Oncogenic (O/LO) Mutations

Considering now only O/LO mutations, the median number of somatic mutations was 4 (range 0–8). More than half of the cohort (37 patients, 54%) could potentially benefit form targeted therapies because of mutations of type *FLT3*-ITD (*n* = 15), *FLT3*-TKD (*n* = 7) or mutations in *IDH1/IDH2 (n* = 20). The number of patients with MR mutations was 40 (58%), 18 of which had more than 1 MR mutation (45%). When we also considered *RUNX1* mutations (as stated in ICC22), the number of affected individuals increased to 46 (67%), with 29 of them (63%) having more than 1 mutation.

Differences in mutation distribution across WHO17 diagnosis (Supplementary Table S2) were only significant for *BCOR/BCORL1*, mostly present in AML-RUNX1m (*p* < 0.001), probably due to their high rate of co-occurrence with *RUNX1* mutations, and for *STAG2*, enriched in AML-MRC (*p* = 0.03). Indeed, only *STAG2* mutations were associated with the presence of MLD, and in particular with dysmegakaryopoiesis.

Eight out of the nine patients with MR-mutations that were considered AML-NOS by WHO17 had at least one mutation in a chromatin modifier gene (*ASXL1*, *n* = 4; *ASXL2*, *n* = 2; *BCOR*, *n* = 3, *EZH2*, *n* = 1, *CREBBP*, *n* = 1, *KDM6A*, *n* = 1), frequently with other MR-mutations. The median number of mutations of this subgroup was higher than that of AML-NOS (4, range 3–7 vs. 2, range 0–6, respectively, *p* < 0.001).

We could not see any differences in the affected pathways or mutation distribution of genes not involved in AML-MR definition. A trend towards a higher presence of *WT1* mutations in AML-DD was detected according to WHO22 (23% of all AML-DD cases vs. 9% of AML-MR, *p* = 0.15). The four *WT1*-mutated cases that belonged to AML-MR presented concomitant mutations in *ASXL1* (*n* = 1), *ASXL1* and *U2AF1* (*n* = 1) or *BCOR* (*n* = 1) or had a history of MDS (*n* = 1). Another two cases with *WT1* mutations also had a *RUNX1* mutation, which is the reason why these cases were evenly distributed between AML-MR and AML-NOS, according to ICC22.

#### 2.2.3. RUNX1 Mutations

**Figure 3:**
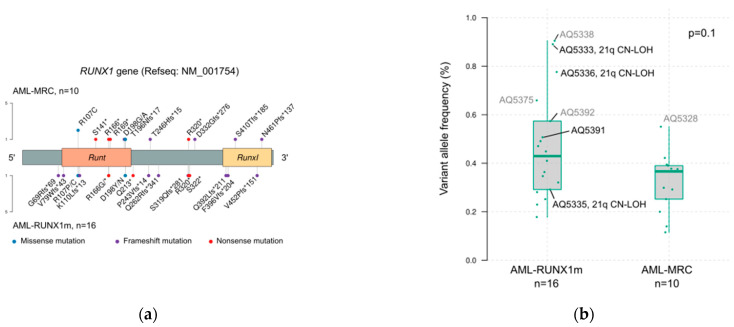
Oncogenic or likely oncogenic *RUNX1* mutations found in 26 patients from the whole cohort. (**a**) Mutation type and location displayed according to WHO17 diagnostic category: AML-MRC (*n* = 10) (upper part) or AML-RUNX1m (lower part); (**b**) variant allele frequency (VAF) distribution of *RUNX1* mutations for each diagnostic category. Cases with VAF > 50% are tagged in black (when copy-number analysis could be done on that sample) or in gray (when the copy-number analysis could not be performed). The three cases with 21q CN-LOH are also marked.

*RUNX1* mutated cases deserved special attention as they have changed the most across new classifications. We detected 37 variants in 27 patients, of which 31 were O/LO. Only one patient presented a single VUS variant. From the 26 patients with at least one *RUNX1* O/LO mutation, 16 were diagnosed with AML-RUNX1m and the other 10 with AML-MRC, because of the presence of MLD (*n* = 8), MDS history (*n* = 6, all with concurrent MLD) or MDS-defining cytogenetics (*n* = 2). Clinical characteristics of patients did not differ between these categories, except for the BM and PB blast count (Table 2). Furthermore, the type of mutation, location within the gene, and variant allele frequency (VAF) were the same for AML-MRC cases and AML-RUNX1m cases (Figure 3).

#### 2.2.4. Pattern of Co-Occurrence and Mutual Exclusivity

**Figure 4:**
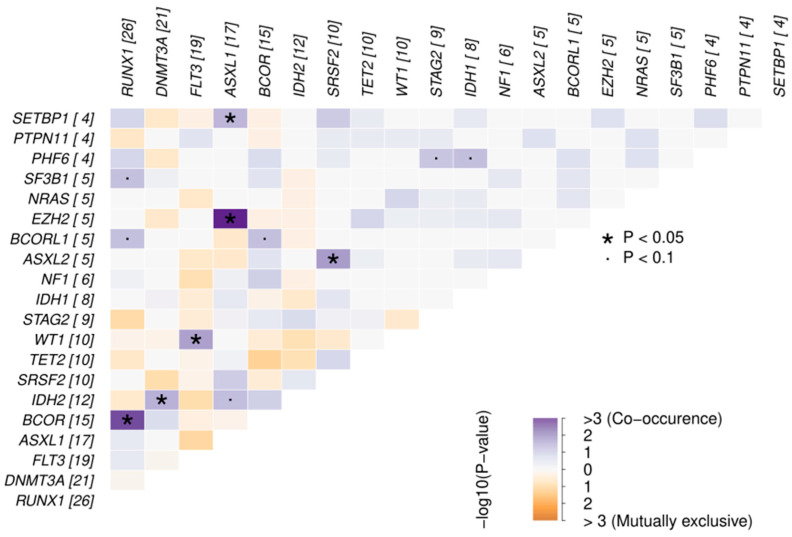
Pattern of co-occurrence and mutual exclusivity of the 20 genes mutated (oncogenic or likely oncogenic variants) in at least four patients from the whole cohort. See in brackets the number of mutated cases for each gene.

We explored the pairwise pattern of co-occurrence and mutual exclusivity of mutations in the 20 genes affected in at least four samples (Figure 4). Chromatin modifiers showed frequent associations with other MR genes (*ASXL1-EZH2*, *p* < 0.001; *ASXL2-SRSF2*, *p* = 0.02) and with myeloid transcription factors (*BCOR-RUNX1*, *p* = 0.002; *ASXL1-SETBP1*, *p* = 0.045). Mutated *RUNX1* also co-occurred frequently with mutations in *BCORL1* (*p* = 0.06) and *SF3B1* (*p* = 0.06). Our cohort also showed other well-known co-occurrence patterns (*FLT3-WT1*, *DNMT3A-IDH2*). In contrast, no significant mutual exclusivity was found between any pair of genes.

#### 2.2.5. Copy-Number Analysis

**Figure 5:**
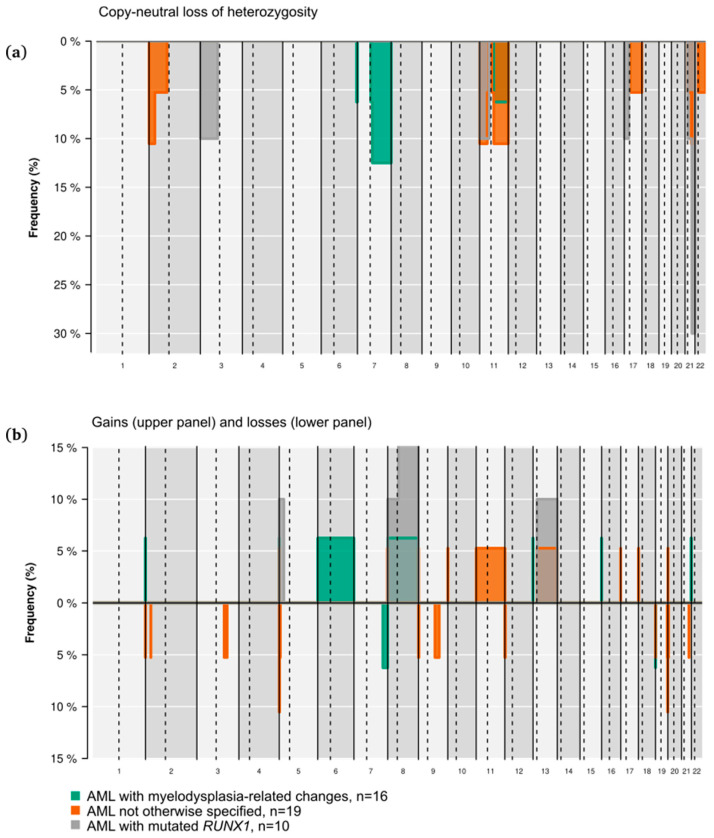
Copy-number alterations of 45 patients with paired tumor-normal DNA. Copy-neutral loss of heterozygosity (**a**) and gains (**b**, upper panel) and losses (**b**, lower panel) in autosomal chromosomes are shown in different colors according to the WHO17 diagnostic category.

We analyzed somatic copy-number alterations (CNAs) in 45 samples with paired tumor-normal DNA. In 28 patients (62%), we found CNAs, being copy-neutral loss of heterozygosity (CN-LOH), the event that affected a higher proportion of patients (42%, 19 patients), in most cases as a single event. CN-LOH events altered a limited subset of eight chromosomes (Figure 5a). In 12 patients (63%), these were associated with mutated genes located in the altered chromosomal region, generally with a VAF > 70% compatible with the homozygous state (Table 3). Only in three cases was VAF < 50%, suggesting that the mutation occurred after CN-LOH.

Gains and losses (shown in Figure 5b) frequently affected a higher number of chromosomal regions from the same patient and/or co-occurred with other CNAs. Trisomies were also restricted to 4 chromosomes (trisomy 8, *n* = 2; trisomy 13, *n* = 2; trisomy 6, *n* = 1 and trisomy 11, *n* = 1). In one case, a focal deletion of 2p involved *DNMT3A* mutation (VAF 91%), and, in another case, loss of 7q affected *EZH2* mutation (VAF of 90%). CNAs were evenly distributed across the different diagnostic categories (Supplementary Tables S2–S4), except for CN-LOH on 21q, which was absent from AML-MRC.

### 2.3. Characteristics of AML-DD/NOS Defined by New Classifications

Sixteen patients are considered AML-DD/NOS by both new classifications. This group is composed of different FAB subtypes (M1, *n* = 6; M2, *n* = 4 and M5, *n* = 6), without finding any significant association to mutations in a particular gene. Median number of mutations was 2 (range 0–6), significantly lower than for AML-MR (*p* < 0.001). Altered genes predominantly belonged to activated signaling pathways, DNA methylation, and tumor suppressors (57, 48, and 38% of samples, respectively).

In particular, there were three patients without recurrently mutated genes included in our NGS diagnostic panel. The three corresponded to middle-aged (range 45–61) women already considered AML-NOS by WHO17 classification. All three had a low leukocyte count at diagnosis (mean WBC 2 × 10^9^/L, range 1.5–2.5). The first patient (AQ5325) corresponded to an M1 FAB subtype with a normal karyotype and without any findings in CNA analysis. She exhibited only a subclonal VUS variant in *CELSFR2* (c.47C>T, p.Pro16Leu; VAF 3%). As reported in a previous work [11], she carries the germ line variant *SDBS* c.258+2T>C in heterozygous state. The second patient (AQ5337) presented with an M5 FAB subtype and showed chromosomal alterations detectable by conventional cytogenetics (trisomy 20) and by CNA analysis (chr3 and chr9 losses). She presented mutations in *EGFR* (c.3533C>T, p.Pro1178Leu; VAF 35%) and *KDM6A*. Her disease’s clinical behavior was very aggressive, being refractory to induction chemotherapy. The third patient (AQ5363) was also a M5 FAB subtype with an unspecific translocation t(8;15)(p13;q22) and a single LO variant in *BCORL1* (c.5264dup, p.Gly1756ArgfsTer4; VAF 21.6%). She also had CN-LOH affecting chr21q without detectable mutations in any gene in this region.

### 2.4. Impact of Mutational Burden on Outcome

When we applied the ELN risk classification, the number of patients falling in the adverse group increased from 41 with 2017 recommendations [12] to 52 with 2022 recommendations [8] due to the addition of MR genes other than *ASXL1* as adverse risk factors. There were no differences in outcomes between both groups with either ELN risk stratification system (Figure S1A,B). Only the presence of >3 O/LO mutations was predictive of a worse survival (*p* = 0.03, Figure S1C). This effect was more obvious when VUS variants were also included, and patients were stratified by the presence of >4 variants (*p* = 0.008, Figure S1D). Looking specifically to the MR genes together with *RUNX1*, a trend to a worse outcome for patients with more than one mutation was observed (*p* = 0.1, Figure S1E).

## 3. Discussion

In this study, we wanted to explore the impact of new classifications on a subgroup of cytogenetic intermediate-risk patients with AML without any of the recurrent genetic abnormalities defined by WHO17. For this purpose, we characterized this cohort through WES by interrogation of 188 genes and a copy-number analysis. Our results are in line with previous studies and confirm their findings [13,14].

All 69 patients had at least one variant and 68 of them had at least one O/LO mutation. Our NGS diagnostic panel covering 40 recurrently mutated genes would have been able to detect at least one mutation in 66 patients (96%). This high percentage and the increase in clinical interpretation uncertainty, when we look at low-frequency, mutated genes support the use of targeted panels in the diagnostic setting, where only variants with high confidence oncogenicity must be taken into account for clinical decision-making. In fact, it has been proposed that a 32-gene panel would be enough to classify all patients with AML [5]. Furthermore, targeted panels offer higher sequencing depth in contrast to ×150 WES, allowing for the detection of minority subclones with VAF as low as 1%, in contrast to the minimal requirement of 5% VAF for variants detected by WES.

In this subgroup of patients, MR mutations are present in as much as 58% of cases, with a high correlation with patients that presented MLD or MDS history (78% and 82% of them have MR mutations, respectively). Our series included only four patients with MR cytogenetic anomalies, two of which also had MR mutations. These results are concordant with the different mutational patterns described for AML-MRC defined by cytogenetics in contrast to AML-MRC defined by MLD or MDS [15]. On the contrary, 21% of patients without any AML-MRC criteria presented MR mutations too. In the seminal study where MR mutations were first described [3], one-third of de novo patients presented these mutations. Much evidence points towards a biological continuum between clonal hematopoiesis, MDS, and AML [16] that would justify the presence of these mutations in the absence of other classic myelodysplasia-defining criteria. We could only find a significant association between *STAG2* and the presence of MLD, in line with previous studies [17]. The limited reproducibility of morphological evaluation, together with this low association to molecular profiles, supports the omission of this criteria in current classifications.

*RUNX1* was the most frequently mutated gene in our cohort. Studies based on a large number of patients revealed differential characteristics from other recurrent genetically defined entities [18], though with some overlap with AML-MRC or AML-NOS. The distribution and location of mutations were comparable between AML-MRC and AML-RUNX1m, as had been already published [19] *RUNX1* mutations co-occurred with other MR-mutations in a high percentage of cases, and specifically with *BCOR/BCORL1* mutations, considerably reducing the impact of the different consideration of this gene in new classifications.

By studying CNA, we could not find any differences between diagnostic categories, which may also be hampered by our limited sample size. CN-LOH was the most prevalent alteration in our series (42% of patients). CN-LOH cannot be routinely assessed by diagnostic laboratories, but it is suggested to improve prognostication, particularly in cytogenetically intermediate-risk AML [20]. Of note, 63% of CN-LOH were associated with mutated driver genes placed in the same affected chromosomal regions. We did not find any 13q CN-LOH, despite it having been identified as the most frequent uniparental disomy (UPD) with a high association with *FLT3* mutations [21,22]. These reports studied cytogenetically normal AML, where a high rate of *NPM1* mutations is expected. This mutation often co-occurs with *FLT3*-ITD and has been specifically associated with UPD [23], therefore we hypothesize that 13q CN-LOH is linked to double mutants *NPM1*/*FLT3*-ITD, a genotype absent in our cohort. Something similar could explain the absence of 6p CN-LOH in our cohort, as it has been only described in *NPM1* mutants [23]. The identification of 2p, 11p, 11q, 17p, and 21q CN-LOH has been frequently described in AML, usually in studies using chromosomal microarrays, although the clinical impact of each specific alteration remains mostly unknown [20]. Nonetheless, the increase in VAF of genes involved in CN-LOH-affected regions supports the hypothesized mechanism of mutation duplication [24]. NGS-targeted panels that include the detection of specific CNA could be a sensible method to investigate clinically meaningful events in the diagnostic setting of AML, even more importantly, when conventional karyotyping results are not available.

Evaluation of prognostic impact was beyond the scope of this work, as the number of patients in this not-uniformly treated series is too small to drive any conclusions. Nonetheless, a higher mutational burden is associated with worse outcomes, as observed before [25], with better accuracy when VUS variants are also considered. A similar trend was detected when OS was analyzed stratifying by one or more than one MR mutation (including *RUNX1*), as has been already described [5,14].

Indeed, even when we focused on a specific subtype of AML with important diagnostic changes, the limited size of our cohort and its genetic complexity precludes having enough statistical power in many comparisons. Despite this, our results are in line with previous studies with larger sample sizes that include all types of AML. Of note, we enriched our work by adding information about low-frequency mutated genes and copy-number alterations.

To conclude, new classifications have an important impact on this group of patients, as 44% of them changed their diagnosis. Mild differences between WHO22 and ICC22 also accounted for diagnostic disparity in 10% of patients. The increase in our knowledge about AML biology is shrinking the AML-DD/NOS hodgepodge, allowing for a better prognostic assignment and therapeutic management. The lower mutational burden of this group suggests the presence of other leukemogenic events, like low-frequency fusion genes or epigenetic mechanisms that will probably be unraveled in the next few years. Our efforts to organize this growing and complex molecular landscape of AML need a hierarchical system with different levels of importance to specific genetic lesions, as current classifications do. It is desirable that forthcoming editions become unified for unambiguous diagnostic categories, owing to their impact on clinical decision-making and patient care.

## 4. Materials and Methods

### 4.1. Patient Selection

Sixty-nine patients with biobanked tumor DNA diagnosed in Hospital Clínic Barcelona between 2000 and 2020 with intermediate risk cytogenetics [26] AML without recurrent genetic abnormalities defined by WHO17 were included. The provisional entity AML with mutated *RUNX1* was not considered as an exclusion criteria, whereas therapy-related AML were excluded. One patient with a hyperdiploid karyotype without structural variants was not considered as having a complex karyotype, as stated in current ELN guidelines.

### 4.2. Sequencing Procedures

DNA from BM (*n* = 57) or PB (*n* = 12) with at least 20% of blasts was extracted with QIAamp DNA Mini Kit (QIAgen). When any of these sources were available, DNA extracted from BM stromal cells (*n* = 28), post-remission BM (*n* = 17), or saliva (*n* = 1) was used as the normal counterpart. WES was performed following an Illumina protocol with ×150 coverage for tumor and ×30 coverage for normal samples. Reads were mapped to human build GRCh38. More details were previously published [11].

### 4.3. Filtering Criteria

Only variants from paired samples with a snpEff predicted annotation impact moderate or high, and at least 8 supporting reads were kept. Variants from samples without paired non-tumoral material were kept when they fulfilled the following criteria: snpEff predicted annotation impact moderate or high, at least 8 reads supporting altered allele, average tumor allele frequency >0.05 and a population allele frequency ≤0.001 (as defined by 1000 genomes project).

We interrogated 188 genes, including 40 recurrently mutated genes included in our NGS diagnostic panel and 148 low-frequency mutated genes reported in the literature [5,25,27,28,29] (see Supplementary Table S5), for which a variant CADD score [30] higher than 15 was required. Selected variants were manually curated according to ClinGen-CGC-VICC joint recommendations [31], excluding benign and likely benign variants. When diagnostic or prognostic impact was analyzed, only oncogenic or likely oncogenic (O/LO) variants were considered.

### 4.4. Copy Number Analysis

Allele-specific copy number analysis was performed using ASCAT (v4.3.0) [32] included in nextflow’s Sarek pipeline (v3.1.1) [33]. Sex chromosomes were discarded from the analysis. Copy number variants were manually curated, excluding low-confidence calls by visual inspection. Genomic Profile v1.0 (https://gclot.shinyapps.io/genomic_profile/, accessed on 6 June 2024) was used for plotting purposes.

### 4.5. Other Tools Used for Genetic Lesion Detection

All *FLT3*-ITD were confirmed by fragment analysis. *KMT2A*-PTD variants and *NUP98* rearrangements were detected by PCR. In 40 cases, information from the diagnostic NGS targeted panel (Oncomine Myeloid Research Assay, Thermo Fisher, Waltham, MA, USA) was available for validation purposes.

### 4.6. Statistical Analysis and Plotting

Associations of continuous measures between two groups were assessed using a Wilcoxon rank-sum test and Kruskal-Wallis test was used for three-group comparisons. Fisher exact test was used to assess associations of categorical variables as well as mutual exclusivity and co-occurrence of O/LO variants. *P* values are unadjusted, 2-sided, and considered significant at 0.05. OS was estimated using the Kaplan-Meier method, the log-rank test for univariate comparison. Maftools package v.2.18.0 [34] was used for data analysis and plotting through R software version 4.3.1 (R core Team, R Foundation for Statistical Computing, Vienna, Austria). Alluvial plots were drawn with package ggalluvial v.0.12.4.

## Figures and Tables

**Table 1:** Clinical characteristics of the total cohort (*n* = 69) and of the three diagnostic groups according to WHO17.

	Total (*n* = 69)	AML-MRC (*n* = 27)	AML-NOS (*n* = 26)	RUNX1m AML (*n* = 16)	*p*
Median age, years (range)	58 (24–81)	61 (24–78)	56 (25–77)	58 (24–81)	0.69
Female sex, n (%)	30 (43)	10 (37)	14 (54)	6 (37)	0.41
WBC (×10^9^/L), median (range)	5.9 (0.5–171)	6.6 (1–171)	5 (0.5–143)	5.7 (0.7–132)	0.93
BM blast count, median (range)	57 (10–98)	39 (10–96)	64 (17–98)	78 (22–91)	**0.002**
PB blast count, median (range)	23 (0–100)	15 (0–92)	30 (0–100)	61 (0–95)	**0.04**
Normal karyotype, n (%)	43 (67)	16 (59)	15 (62)	12 (92)	0.09
ELN22 defined adverse risk, n (%)	51 (74)	23 (85)	12 (46)	16 (100)	NA
AML-MRC criteria					
Previous MDS, n (%)	11 (16)	11 (39)	0	0	NA
Morphologic dysplasia, n (%)	23 (33)	23 (82)	0	0	NA
MDS-defining cytogenetics, n (%)	4 (6)	4 (15)	0	0	NA
Treatment received					
Intensive induction chemotherapy, n (%)	59 (85)	22 (81)	22 (85)	15 (94)	0.62
Allogeneic HCT, n (%)	42 (61)	15 (55)	15 (62)	12 (92)	0.42
Autologous HCT, n (%)	5 (7)	1 (4)	3 (11)	1 (6)	0.73
Disease response after induction (*n* = 58)					
Complete response, n (%)	52 (90)	18 (82)	19 (86)	14 (93)	0.87
Refractory disease, n (%)	6 (10)	3 (13)	2 (9)	1 (7)	0.87
Early death, n (%)	2 (3)	1 (4)	1 (5)	0	1

Abbreviations: AML, acute myeloid leukemia; MRC, myelodysplasia-related changes; NOS, not otherwise specified; RUNX1m, with *RUNX1* mutation; WBC, white blood cell count; BM, bone marrow; PB, peripheral blood; MDS, myelodysplastic syndrome, HCT, hematopoietic cell transplantation; NA, not applicable; ELN22, European LeukemiaNet risk stratification [8].

**Table 2:** Clinical characteristics of patients with mutations in *RUNX1* according to diagnosis (*n* = 26).

	Total (*n* = 26)	AML-MRC (*n* = 10)	RUNX1m AML (*n* = 16)	*p*
Median age, years (range)	58 (24–81)	65 (41–78)	58 (24–81)	0.19
Female sex, n (%)	9 (35)	3 (30)	6 (37)	1
WBC (×10^9^/L), median (range)	7.8 (0.7–134)	12.7 (1.7–134)	5.7 (0.7–132)	0.34
BM blast count, median (range)	67 (21–91)	49 (21–81)	78 (22–91)	**0.02**
PB blast count, median (range)	27 (0–95)	16 (0–72)	61 (0–95)	**0.04**
Cytogenetics (*n* = 65)				
Normal karyotype, n (%)	18 (69)	6 (60)	12 (92)	0.13
Number of variants *, median (range)	6 (1–12)	5.5 (3–10)	6 (1–12)	0.59
Number of mutations **, median (range)	5 (1–7)	4.5 (3–7)	5 (1–7)	0.5
Myelodysplasia-related and *RUNX1* mutations **, median (range)	1 (0–3)	1 (0–3)	1 (0–2)	0.3
*RUNX1* mutations (*n* = 31)				
Missense variant	9	4	5	1
Frameshift/Nonsense variant	22	9	13	1
Multi-hit	5	3	2	NA
Chr21q23 CN-LOH	3	0	3	NA
Variant allele frequency (mean, range)	0.4 (0.11–0.9)	0.32 (0.11–0.55)	0.47 (0.18–0.9)	0.1

* Includes oncogenic, likely oncogenic and uncertain significance variants. ** Includes only oncogenic and likely oncogenic variants. NA, not applicable.

**Table 3:** Copy-neutral loss of heterozygosity events found in 45 evaluated patients, and the mutations detected in the affected chromosomal regions.

Sample ID	Chromosome	Start (GRCh38)	End (GRCh38)	Gene	HGSVc	HGSVp	VAF (%)
AQ5342	chr2 (p25.3–p11.2)	41,404	85,325,063	*DNMT3A*	c.1742G>A	p.Trp581Ter	71
AQ5327	chr2 (p25.3–p23.3)	41,404	27,616,502	*DNMT3A* *	c.1813del	p.Leu605SerfsTer46	97
AQ5366	chr6 (q27)	166,931,095	170,583,760	NA			
AQ5357	chr7 (q11.21–q36.3)	63,096,280	159,232,490	*EZH2*	c.203_204del	p.Val68AlafsTer13	85
AQ5390	chr7 (q11.22–q36.3)	70,768,153	159,232,490	*CUX1*	c.634C>T	p.Gln212Ter	98
AQ5368	chr11 (p15.5–p11.2)	43,754,184	43,554,371	NA			
AQ5359	chr11 (p15.5–p11.2)	199,813	45,812,493	*WT1*	c.1152dup	p.Arg385ThrfsTer5	79
AQ5344	chr11 (p15.5–p13)	199,813	35,968,505	*WT1* **	c.1264+1G>C		73
AQ5340	chr11 (q13.2–q25)	66,551,501	134,857,757	*KMT2A*-PTD			NA
AQ5380	chr3 (p26.3–p12.2)	319,825	81,648,979	NA			
AQ5383	chr11 (q12.1–q25)	57,688,772	134,857,757	*CBL*	c.1228-1G>A		88
AQ5389	chr11 (q13.1–q25)	65,999,744	134,857,757	*KMT2A*-PTD			NA
AQ5351	chr17 (q11.1–q25.3)	27,280,695	83,054,873	*NF1*	c.4577+2T>G		86
AQ5395	chr17 (p13.3–p11.2)	161,952	21,016,024	NA			
AQ5335	chr21 (q11.2–q22.3)	13,384,722	46,608,083	*RUNX1*	c.592G>T	p.Asp198Tyr	29
AQ5363	chr21(q11.2–q22.3)	13,384,722	46,608,083	NA			
AQ5331	chr21 (q21.3–q22.3)	29,591,425	46,608,083	***			
AQ5333	chr21 (q22.11–q22.3)	32,268,841	46,608,083	*RUNX1*	c.496C>T	p.Arg166Ter	89
AQ5336	chr21 (q22.11–q22.3)	32,319,444	46,618,727	*RUNX1*	c.637C>T	p.Gln213Ter	78
AQ5351	chr22 (q11.1–q13.33)	16,136,750	50,740,572	NA			

* The same patient also had mutations in ASXL2 with VAF < 50%, although this gene is encom-passed in the 2p CN-LOH region. ** The same patient also had a second mutation in WT1 (c.812dup, p.Val272GlyfsTer26) with VAF 12%. *** Homozygous copy loss detected in RUNX1 locus (q22.12). NA, not applicable.

## Data Availability

Sequencing data are available from the National Center for Biotechnology Information Sequence Read Archive under accession number PRJNA994311.

## Supplementary Materials

The following supporting information can be downloaded at: https://www.mdpi.com/article/10.3390/ijms25168669/s1.
