# Supplementary material for: Whole Exome Sequencing of Intermediate-Risk Acute Myeloid Leukemia without Recurrent Genetic Abnormalities Offers Deeper Insights into New Diagnostic Classifications
Source: Int J Mol Sci. 2024 Aug 8;25(16):8669. doi: 10.3390/ijms25168669 (PMC11354381; doi:10.3390/ijms25168669)
Supplement: Supplementary file 1 [file ijms-25-08669-s001.zip › ijms-3087068-supplementary/Suppl_Figure S1.pdf]

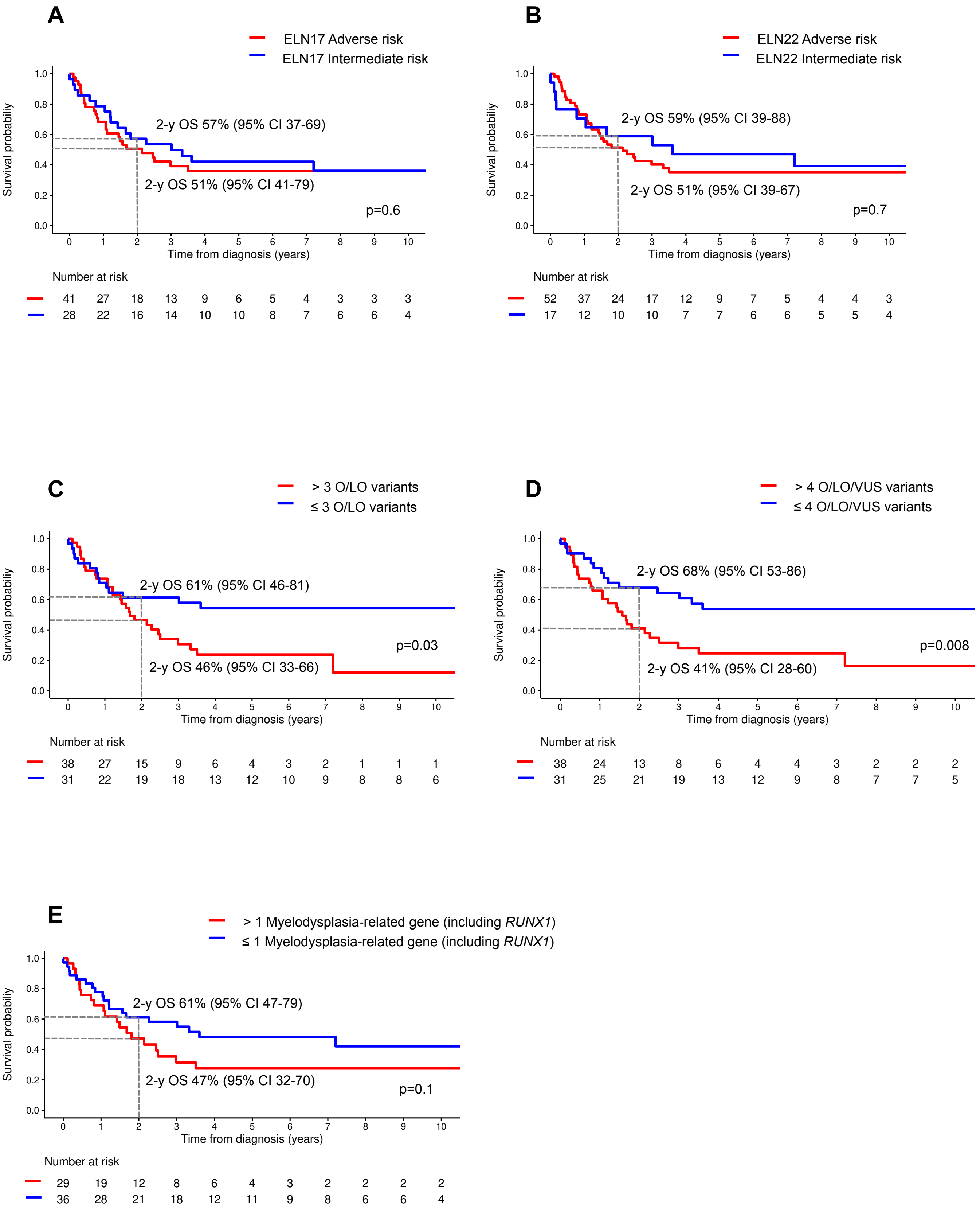

Figure S1: Overall survival from the whole cohort (n=69) stratified by European LeukemiaNet 2017 risk classification (A), European LeukemiaNet 2022 risk classification (B), presence of more than 3 oncogenic/likely oncogenic mutations (C), presence of more than 4 variants, including those of unknown significance, likely oncogenic and oncogenic variants (D). Last plot (E) shows overall survival of 65 patients (excludes the patients with AML with *NUP98* rearrangements (n=3) and the patient with AML with *TP53* mutation, n=1) stratified by the presence of more than one mutation (only oncogenic/likely oncogenic variants) affecting *ASXL1, BCOR, EZH2, RUNX1, SF3B1, SRSF2, STAG2, ZSRS2* or *U2AF1*.
